# Microbiome-Assisted Breeding to Understand Cultivar-Dependent Assembly in *Cucurbita pepo*

**DOI:** 10.3389/fpls.2021.642027

**Published:** 2021-04-09

**Authors:** Peter Kusstatscher, Eveline Adam, Wisnu Adi Wicaksono, Maria Bernhart, Expedito Olimi, Henry Müller, Gabriele Berg

**Affiliations:** ^1^Institute of Environmental Biotechnology, Graz University of Technology, Graz, Austria; ^2^Saatzucht Gleisdorf GmbH, Gleisdorf, Austria

**Keywords:** pumpkin, *Cucurbitaceae*, plant–microbe interactions, 16S rRNA gene amplicons, ITS sequencing, microbiome transmission

## Abstract

Recently, it was shown that long-term plant breeding does not only shape plant characteristics but also impacts plant-associated microbiota substantially. This requires a microbiome-integrative breeding approach, which was not yet shown. Here we investigate this for the Styrian oil pumpkin (*Cucurbita pepo* L. subsp. *pepo* var. *styriaca* Greb.) by analyzing the microbiome of six genotypes (the complete pedigree of a three-way cross-hybrid, consisting of three inbred lines and one open pollinating cultivar) in the seed and rhizosphere as well as the progeny seeds. Using high-throughput amplicon sequencing targeting the 16S rRNA and the ITS1 genes, the bacterial and fungal microbiomes were accessed. Seeds were found to generally carry a significantly lower microbial diversity compared to the rhizosphere and soil as well as a different microbial composition, with an especially high fraction of *Enterobacteriaceae* (40–83%). Additionally, potential plant-beneficial bacterial taxa, including *Bacillaceae*, *Burkholderiaceae*, and *Pseudomonadaceae*, were found to be enriched in progeny seeds. Between genotypes, more substantial changes can be observed for seed microbiomes compared to the rhizosphere. Moreover, rhizosphere communities were assembled for the most part from soil. Interestingly, bacterial signatures are mainly linked from seed to seed, while fungal communities are shaped by the soil and rhizosphere. Our findings provide a deep look into the rhizosphere and seed microbiome assembly of pumpkin-associated communities and represent the first steps into microbiome-driven breeding for plant-beneficial microbes.

## Introduction

Microbes play a key role in plant development and health throughout the whole life cycle (Mendes et al., 2011; Philippot et al., 2013; Vandenkoornhuyse et al., 2015). Therefore, it is important to identify all influencing factors as well as sources of the plant microbiota (Berg et al., 2016; Cordovez et al., 2019). The impact of plant genotype and soil quality on the diversity of the rhizosphere microbiome was studied for decades now (Smalla et al., 2001; Berg and Smalla, 2009; Lundberg et al., 2012). The influence of breeding on the microbiome, however, was just recently identified (Peiffer and Ley, 2013; Bouffaud et al., 2014; Cardinale et al., 2015). Another recent discovery was shown for the seed microbiome; plant-associated microorganisms, including plant-beneficial microbes, are transferred *via* seed into the next generation (Johnston-Monje et al., 2016; Adam et al., 2018; Bergna et al., 2018). Interestingly, genotype-specific microbial communities are even more pronounced in seeds (Rybakova et al., 2017; Adam et al., 2018; Chen et al., 2020). Taken together, the knowledge of transferring core microbiomes from one generation to the other (Berg and Raaijmakers, 2018), which can be influenced by targeted breeding (Mendes et al., 2019), opens new possibilities for innovative plant breeding and protection strategies (Cordovez et al., 2019). They are urgently needed to ensure food security despite climate change, biodiversity loss, and emerging pathogens, but not yet exploited.

The Styrian oil pumpkin (*Cucurbita pepo* L. subsp. *pepo* var. *styriaca* Greb.) represents a relatively new *Cucurbitaceae* derived from the Austro-Hungarian Empire in the nineteenth century. In contrast to other *C. pepo* varieties, the Styrian oil pumpkin lacks lignification of the seed coat, which makes it suitable for oil extraction. The dark-green oil made from the seeds is traditionally consumed in Austria and found its way into international gourmet cuisines. The oil is rich in polyunsaturated fatty acids and contains vitamins, polyphenols, minerals, and phytosterols (Fruhwirth and Hermetter, 2007). The unique pumpkin cultivar is, with an acreage of over 35,000 ha, one of the mainly grown crops in southern Austria, and additional growing areas are established in Africa, China, and Eastern Europe (Estyria, 2018). However, the lack of lignification of the seed coat also leads to a high susceptibility to various fungal and bacterial diseases during seed germination (Heinisch and Ruthenberg, 1950). The seeding of untreated seeds frequently ends in huge losses due to seed infections; therefore, commercial seeds are treated with chemical strippers such as copper-based or synthetic fungicides. The Styrian oil pumpkin is a good model to study beneficial plant–microbe interactions during breeding because the breeding history is short and well known, seed and pumpkin microbiomes were already studied, and main pathogens causing dumping off, fruit rot, viruses, and leaf diseases are identified (Winkler et al., 2008; Grube et al., 2011; Bedlan, 2012; Pachner et al., 2015; Adam et al., 2018). Moreover, novel disease-resistant cultivars and potential key antagonists are important to reduce the dependence of pumpkin grown on chemicals.

Our study aims to disentangle the effect of genotypes on bacterial and fungal communities in seeds and in the rhizosphere of oilseed pumpkin. Therefore, we studied six genotypes (the complete pedigree of a three-way cross-hybrid, consisting of three inbred lines and one open pollinating cultivar) by analyzing 16S rRNA and ITS gene amplicon libraries. We assessed differences between genotypes in seed and rhizosphere and compared seeds sown and their progeny seeds to observe microbiome shifts induced by propagation. By tracking microbial transmission from seeds, soil, and rhizosphere of the different genotypes, the impact of breeding on seed colonization is evaluated, and new insights from a breeder’s and microbiologist’s perspective, respectively, are generated.

## Materials and Methods

### Pumpkin Genotypes

For this study, the mainly used cultivar in organic farming (“GL Classic”) and the most prevalent cultivar in conventional farming (“GL Rustikal”) as well as its pedigree components were chosen. Seeds of inbred lines A, B, and D as well as cross-hybrids ‘Gl. Diamant’ (A × B), ‘GL Rustikal’ (AB × D), and the open pollinating cultivar ‘GL Classic’ were provided by Saatzucht Gleisdorf GmbH. Production of the seeds used in the experiments was done on two field sites in Gleisdorf (province of Styria, Austria) in 2014 (Table 1). Post-harvest processing in seed production was performed according to the standard procedures of the Saatzucht Gleisdorf GmbH breeding station by washing the seeds with water directly after harvest and drying them at a temperature of maximum 40°C down to a moisture content of 8%.

**TABLE 1:** Characteristics of *Cucurbita pepo* genotypes selected for the microbiome analysis.

Denomination	Category	Pedigree	Field site origin of seeds
Line A	Inbred line	–	47°06′46.0″ N, 15°41′57.9″ E (Pfarrhoffeld)
Line B	Inbred line	–	47°06′46.0″ N, 15°41′57.9″ E (Pfarrhoffeld)
Line D	Inbred line	–	47°06′46.0″ N, 15°41′57.9″ E (Pfarrhoffeld)
Gl. Diamant	Single cross-hybrid	Line A × line B	47°07′03.6″ N, 15°42′22.8″ E (Teichacker)
GL Rustikal	Three-way cross-hybrid	Gl. Diamant × line D	47°07′03.6″ N, 15°42′22.8″ E (Teichacker)
GL Classic	Open-pollinated cultivar	–	47°06′46.0″ N, 15°41′57.9″ E (Pfarrhoffeld)

### Field Experiments and Sampling Strategy

Seeds were planted in a field at the breeding station of Saatzucht Gleisdorf GmbH (47°06’55.1″ N 15°42’28.9″ E). A total of 40 seeds per genotype were coated with 0.3 g of the fungicide Maxim^®^ XL (Syngenta) and split into four replicates. The soil of the field sites is described as gleyed loose brown earth, loamy silt, and cover loams on a quaternary terrace deficient in lime, with a pH value of 6.5. Rhizosphere samples were taken from four randomly chosen plants per replicate at 1 month after sowing (phenological growth stage inflorescence emergence, BBCH: 52). The soil around a plant was loosened with a spade, and the root system was exposed. Parts of the primary and secondary roots as well as of fine roots were pooled to one rhizosphere sample per plant. Additionally, four soil samples were also taken from the field site at random locations in the free place between the replicate plots with approximately 80 cm distance to the plants at the same depth where the main parts of the root system was located at that stage (5 to 15 cm). Five to 7 g of each rhizosphere and soil replicate was suspended in 50 ml 0.85% NaCl and homogenized by a 3-min bag mixer (stomacher) treatment; then, 4 ml of the homogenized solution was pelleted for 20 min at 4°C and 13,500 *g*.

Additionally, a total of 40 seeds of each genotype (original seeds sown) were washed five times and soaked in 25 ml sterile deionized water for 4 h at 100 rpm. Seeds were divided into four replicates (10 seeds each) and grounded with a pestle in 10 ml 0.85% NaCl in a sterile bag (Nasco Whirl-Pak^®^). A total of 3 ml suspension was pelleted as described above. The same procedure was performed with seeds harvested in the course of the field experiment (progeny seeds). Briefly, pumpkin fruits were washed and opened with a sterile knife. The seeds were extracted carefully using clean gloves, washed with water, and dried separately per fruit. Then, seeds of at least four fruits per replicate plot were pooled to a sample of 10 seeds per plot and processed as described above. A total of 76 samples [four replicates per sample type (seed, progeny seeds, and rhizosphere) and genotype (*N* = 6); four soil samples] were prepared for further analysis.

### DNA Extraction and Amplicon Sequencing

Total DNA was extracted from all samples using FastDNA^TM^ SPIN Kit for Soil (MP Biomedicals, Santa Ana, CA, United States) with a slightly modified protocol. DNA samples were quality-checked using Nanodrop 2000 (Thermo Fisher Scientific, Wilmington, DE, United States) and stored at −20°C for further PCR reactions.

Using a targeted amplicon strategy, the bacterial and fungal microbiomes were assessed. The 515f/806r primer pair (515f: 5′-GTGYCAGCMGCCGCGGTAA-3′; 806r: 5′-GGACTACNVGGGTWTCTAAT-3′) targeting the 16S rRNA gene V4 hypervariable region (Caporaso et al., 2011; Parada et al., 2016) and the ITS1f/ITS2 primer pair (ITS1f: 5′-CTTGGTCATTTAGAGGAAGTAA-3′; ITS2r: 5′-GCTGCGTTCTTCATCGATGC-3′) targeting the ITS1 region (White et al., 1990) were used. All PCR reactions were performed in triplicate. The 16S rRNA gene amplification was performed in 30 cycles at 96°C denaturation for 60 s, 78°C peptide nucleic acid (PNA) annealing for 5 s, 54°C primer annealing for 60 s, and 74°C elongation for 60 s. Peptide nucleic acid PCR clamps were used to block the amplification of plastids and mitochondrial 16S rRNA gene sequences. For blocking of pumpkin DNA in ITS amplification, a customized PCR clamp was designed. ITS amplicons were performed in a two-step PCR approach. The first PCR step was performed using ITS primer with an attached linker (to attach barcodes). Following a 5-min initial denaturation at 30 cycles of 95°C denaturation for 30 s, 78°C PNA annealing for 5 s, 58°C primer annealing for 35 s, and 72°C elongation for 40 s were performed. The second PCR step was 15 cycles of 95°C for 30 s, 53°C for 30 s, and 72°C for 30 s using the first PCR as template and attaching individual barcode sequences for each sample. The PCR products were purified using Wizard SV Gel and PCR Clean-Up System (Promega, Madison, WI, United States) and pooled in a 16S and ITS pool with equimolar concentrations. The barcoded Illumina libraries were sent for paired-end Illumina MiSeq sequencing (2 × 300 bp, GATC Biotech, Berlin, Germany). The obtained 16S rRNA and ITS amplicon raw reads were deposited at the European Nucleotide Archive under project number PRJEB41779.

### Bioinformatic Data Processing

Quality-checked sequences were demultiplexed using cutadapt (Martin, 2011). The DADA2 algorithm in QIIME2 was used to generate representative sequences and a feature table (Callahan et al., 2016; Bolyen et al., 2019). Taxonomic classification was performed using vsearch algorithm and SILVA v132 and UNITE v8 databases as bacterial and fungal references (Kõljalg et al., 2013; Quast et al., 2013; Rideout et al., 2014; Nilsson et al., 2019). Non-target sequences (chloroplasts, mitochondria, and Archaea) were removed prior to further statistical analyses. The sourcetracker2 software was used to identify community linkage between the sample types of each genotype (Knights et al., 2011).

### Statistical Analysis

R version 1.2.1335 (Allaire, 2012; R Core Team, 2013) was used to perform statistical analysis and create graphs unless stated otherwise. Feature table and taxonomic information were exported and further analyzed using the Phyloseq package (McMurdie and Holmes, 2013). Prior to the alpha and beta diversity analysis, each dataset was normalized to the lowest number of read counts by randomly selecting subsets of sequences (*N* = 485 and 300 for bacterial and fungal datasets, respectively). The alpha diversity was calculated using Shannon diversity index. Prior to analysis of variance (ANOVA), a normality test was performed using Shapiro test. If the data were not normally distributed, the non-parametric Kruskal–Wallis test was used instead of ANOVA. The beta diversity analysis was assessed using normalized Bray–Curtis dissimilarity matrix and then subjected to permutational analysis of variance (PERMANOVA, 999 permutations). Principal coordinate analysis plots were generated to visualize the clustering of bacterial and fungal communities according to sample type and genotype.

## Results

### Bacterial and Fungal Diversity in Seeds, Soil, and Rhizosphere

**FIGURE 1:**
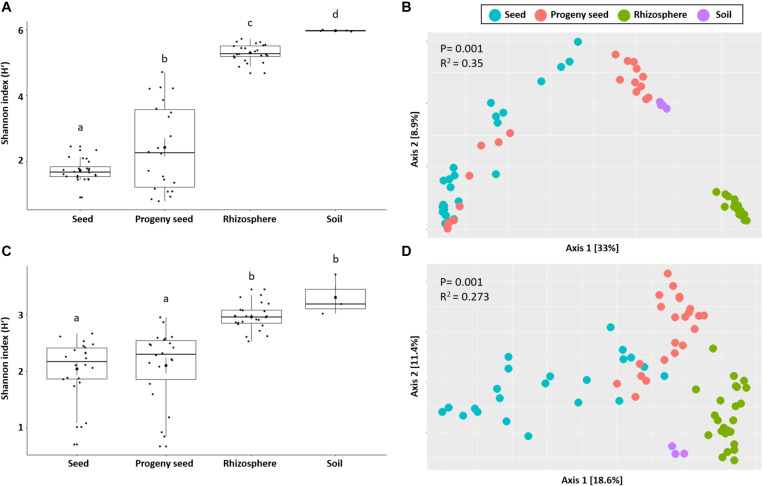
Observed bacterial **(A,B)** and fungal **(C,D)** diversity in samples. Shannon index (alpha diversity) as well as beta diversity obtained by Bray–Curtis distance matrix is shown. Significant differences are indicated by different letters as well as given *p*-values.

After quality filtering and removal of non-target taxa (chloroplast, mitochondrial DNA, and archaeal sequences), a total of 14,259,303 and 591,998 high-quality reads were retained from bacterial and fungal datasets, respectively (Supplementary Table 1). By applying the DADA2 algorithm, the bacterial and fungal reads were clustered into 32,221 and 780 amplicon sequence variants, respectively. Analyzing the alpha diversity of seed, rhizosphere, soil, and progeny seed samples, overall major differences were observed. Soil generally carried the highest Shannon diversity index [mean *H*’ = 6.0 (bacteria) and *H*’ = 3.3 (fungi)], followed by rhizosphere samples [mean *H*’ = 5.3 (bacteria) and *H*’ = 3.0 (fungi)]. Sown seed and progeny seed samples carried the lowest diversity [mean *H*’ = 1.7 and 2.4 (bacteria) as well as *H*’ = 2.0 and 2.1 (fungi)]. Overall significant differences (*p* < 0.001) were found for all sample types in bacterial diversity measures. Fungal diversities were different between belowground (rhizosphere and soil) and seed samples, however not within those groups (Figures 1A,C). Moreover, beta diversity analysis showed a clear clustering between seed samples, soil, and rhizosphere from both community datasets. PERMANOVA analysis demonstrated that sample type significantly affected bacterial and fungal community structure (*p* = 0.001). This factor explained 35 and 27% of bacterial and fungal community variation, respectively (Figures 1B,D).

**FIGURE 2:**
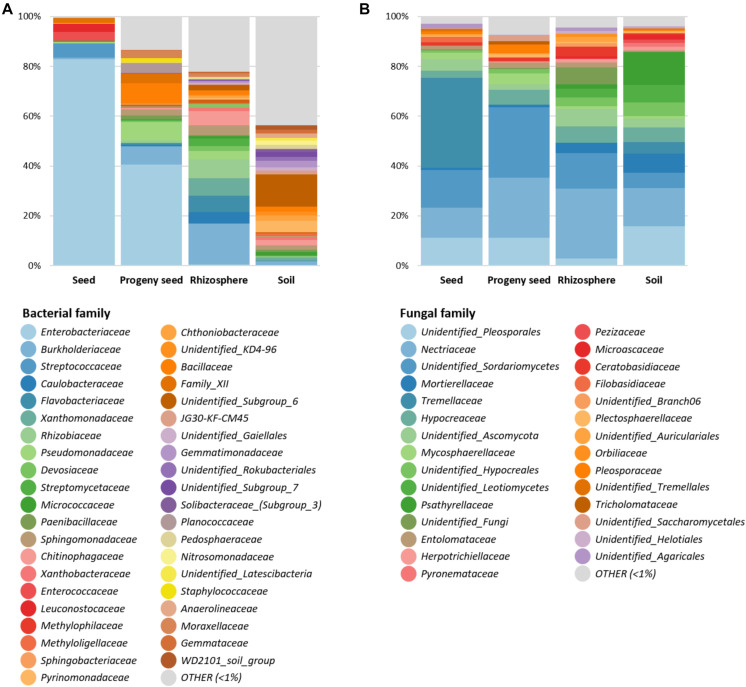
Relative taxonomic composition of four sample types (seed, progeny seed, rhizosphere, and soil). Bacterial **(A)** and fungal **(B)** taxa on family level with abundance higher than 1% are shown individually. Low-abundance taxa are summarized as “OTHER.”

After feature classification, the most abundant features (> 1% relative abundance) were visualized to reduce the complexity (Figure 2). Overall, a clear difference in obtained bacterial and fungal microbiomes was observed for all sample types. In general, *Proteobacteria* (67.9%) was the most predominant bacterial phylum, followed by *Firmicutes* (12.4%) and *Bacteroidetes* (6.5%). While seeds and progeny seeds were mainly colonized by *Enterobacteriaceae* (82.8 and 40.5%, respectively), the main observed families in the rhizosphere were *Burkholderiaceae* (16.4%), *Rhizobiaceae* (7.5%), and *Flavobacteriaceae* (6.5%). In the soil, generally, a higher diversity was observed, with numerous bacterial families (*n* = 25) distributed evenly in a range between 1 and 4% of total relative abundance. Taxa with a higher abundance were “unidentified Subgroup_6” (12.9%) and *Pyrinomonadaceae* (4.6%). A total of 43.6% of observed taxa were below 1% relative abundance and therefore classified as other (Figure 2A).

In the fungal dataset, *Ascomycota* and *Basidiomycota* were the dominant fungal phyla and accounted for 73.1 and 21.9% of total community, respectively. Seeds were mainly colonized by the families *Tremellaceae* (36.2%) and *Nectriaceae* (12.2%) and an unidentified *Sordariomycetes* taxon (15.1%). Progeny seeds were mainly colonized by unidentified *Sordariomycetes* taxa (28.1%), *Nectriaceae* (24%), and unidentified *Pleosporales* taxa (11.3%). The rhizosphere was mainly colonized by *Nectriaceae* (28%), unidentified *Sordariomycetes* (14.3%), and *Hypocreaceae* (6.8%). The soil showed a higher taxonomic diversity and was mainly colonized by an unidentified *Pleosporales* taxa (15.7%), *Nectriaceae* (15.4%), *Psathyrellaceae* (13.3%), and *Mortierellaceae* (7.7%) (Figure 2B).

### Genotype-Specific Differences in Seeds, Rhizosphere, and Progeny Seeds

The genotype affected bacterial and fungal beta diversity, but it was not statistically significant for the alpha diversity in seeds sown. In general, GL Rustikal [(A × B) × D] carried a higher bacterial alpha diversity (*H*’ = 2.1) compared to other genotypes (*H*’ = 1.5–1.8). On the other hand, line B carried a higher fungal alpha diversity (*H*’ = 2.4) compared to other genotypes (*H*’ = 1.5–2.3). However, the differences were only statistically significant for bacteria (*p* = 0.039 and *p* = 0.221 for bacterial and fungal datasets, respectively). Beta diversity showed a significant clustering of samples in both the bacterial and fungal datasets (*p* = 0.001) (Supplementary Figure 1). The genotype effect explained 72 and 43% of variance between samples for bacteria and fungi, respectively. In addition, a separate beta diversity analysis indicated that the field origin of sown seeds also affected the bacterial and fungal community structures (*p* = 0.001). The field origin explained 17 and 19% of variance between samples for bacteria and fungi, respectively (Supplementary Figure 2). The soil analysis of those fields showed a slight variation in soil characteristics (Supplementary Table 2). The genotype effect was less pronounced for progeny seeds. No significant differences were found for alpha and beta diversity for both bacterial and fungal communities in progeny seed (*p* > 0.05). However, the genotype still explained 22 and 29% of diversity between samples for bacteria and fungi, respectively (Supplementary Figure 3).

**FIGURE 3:**
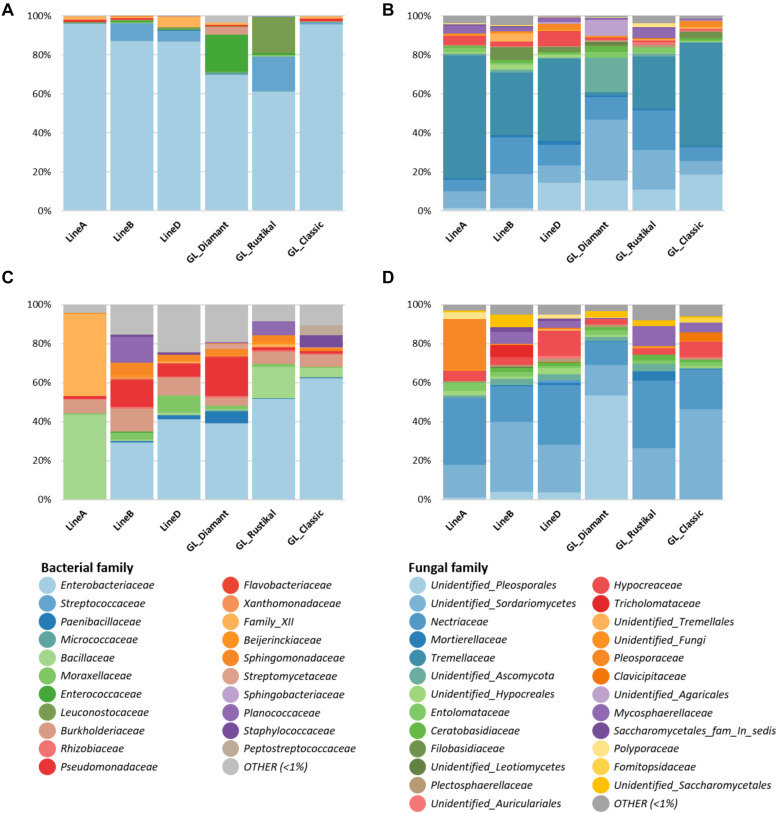
Relative taxonomic composition of seeds from different genotypes [sown seeds **(A,B)** and progeny seeds **(C,D)**]. Bacterial **(A,C)** and fungal **(B,D)** taxa above 1% relative abundance on family level are shown. Low-abundance taxa (< 1%) are summarized as “OTHER.”

The observed differences in diversity are also reflected in the found taxonomic compositions of the seeds and progeny seeds. In seeds sown, despite a generally high occurrence of *Enterobacteriaceae* as previously described, GL Rustikal [(A × B) × D] carried relatively higher proportions of *Streptococcaceae* (17.7%) and *Leuconostocaceae* (18.5%) in comparison to the other genotypes (0–9.2 and < 1.1%, respectively). Moreover, Gl. Diamant (A × B) carried a relatively higher abundance of *Enterococcaceae* (7.5%) compared to others (< 1.2%). The progeny seeds overall showed a higher taxonomic diversity. They were also colonized by a high abundance of *Enterobacteriaceae* except for line A, which showed mainly *Bacillaceae* (43.6%), *Family XII* (42%), and *Burkholderiaceae* (7.3%). Additionally, a higher proportion of *Pseudomonadaceae* was found in line B (13.6%) and Gl. Diamant (A × B) (19.4%) in comparison to others (< 6.2%). The families *Planococcaceae*, *Burkholderiaceae*, *Sphingomonadaceae*, and *Bacillaceae* were further also found in higher abundances in multiple genotypes (Figures 3A,C).

An analysis of the fungal taxonomic composition of sown seeds indicated that line A and GL Classic harbored a relatively higher *Tremellaceae* fraction (63 and 53%, respectively) in comparison to other pedigree lines (1.7–42.1%). In contrast, these two genotypes harbored a lower unidentified *Sordariomycetes* fraction (8.5 and 7.1%) in comparison to other genotypes (8.9–31%), of which the highest proportion of unidentified *Sordariomycetes* was detected in Gl. Diamant (A × B). Fungal families such as *Pleosporales* and *Nectriaceae* were further of higher abundance in all genotypes. In the fungal community of progeny seeds, Gl. Diamant (A × B) again showed a distinguishable microbial composition from the other cultivars, with a high proportion of unidentified *Pleosporales* (53.5%). Progeny seeds from GL Classic harbored a relatively higher unidentified *Sordariomycetes* (46.4%) in comparison to others (13.5–35.9%). Line A showed a relatively high proportion of *Pleosporaceae* (25.4%), which was not found in other genotypes. Additionally, *Nectriaceae* and *Hypocreaceae* were fungal taxa found in higher abundance in all progeny seeds (Figures 3B,D).

The rhizosphere microbiome of the different genotypes was, in contrast to the seeds, relatively uniform. The alpha bacterial and fungal diversities were not significantly different between genotypes (*p* = 0.566 and *p* = 0.679, respectively). Although the genotype explained 23% of both bacterial and fungal community variation, this factor did not significantly affect the bacterial and fungal beta diversity (*p* = 0.271 and *p* = 0.466, respectively). Moreover, a relatively similar bacterial and fungal taxonomic composition was observed in the rhizosphere samples, and only minor genotype effects are visible (Supplementary Figure 4).

### Microbiome Assembly Along the Pedigree and in the Rhizosphere and Progeny Seeds

**FIGURE 4:**
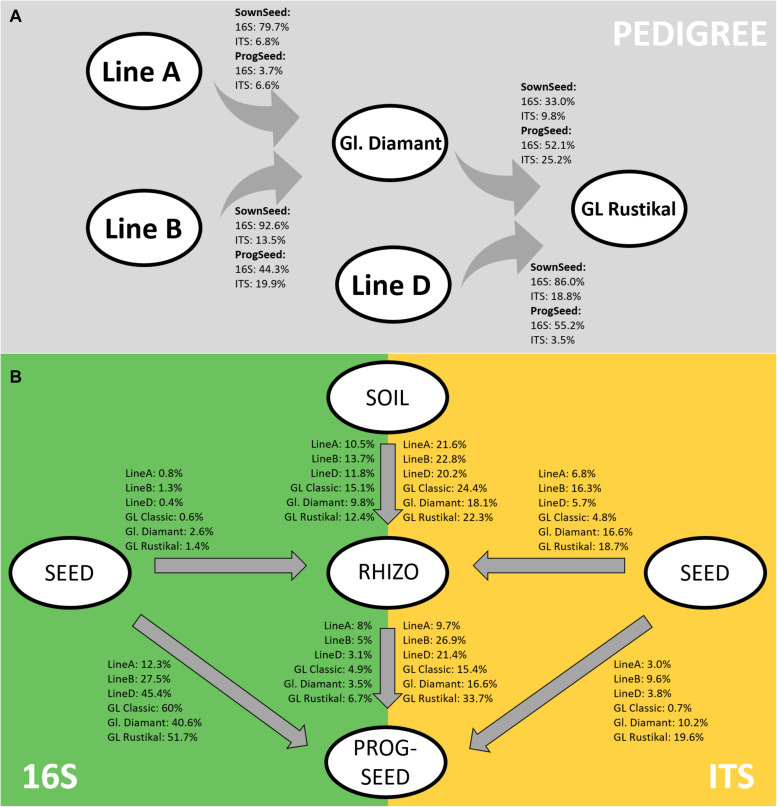
Tracking of bacterial and fungal microbiomes across the pedigree in sown seeds and progeny seeds **(A)** as well as from sown seeds and soil to rhizosphere and progeny seeds **(B)** using sourcetracker2. Ident bacterial (16S) and fungal (ITS) traces are indicated as percentage.

Using the sourcetracker2 software, microbiome similarities were further analyzed along the pedigree. Both the sown seeds and progeny seeds were analyzed (Figure 4A). In the sown seeds, high bacterial fractions (33–93%) were shared between the hybrid lines and their parental components. In contrast, fungal traces were shared to a lesser extent (7–19%). Interestingly, the assembly of the microbiome of GL Rustikal was more influenced by line D (86 and 19% for bacterial and fungal traces, respectively) as by Gl. Diamant (33 and 10% for bacterial and fungal traces, respectively). Similarly, in the progeny seeds, higher fractions were generally shared in the bacterial microbiome, compared to the fungal fraction, even though fungi were shared by up to 25%. One exception was observed with line A, which did not share much with Gl. Diamant (3.7 and 6.6% for the bacterial and fungal fraction, respectively).

Moreover, the link of microbial communities between sown seeds to the rhizosphere and their progeny seeds was tracked. The highest percentage of bacterial linkage was found from sown seeds to progeny seeds, while fungi were mostly linked between soil and rhizosphere as well as rhizosphere and progeny seeds (Figure 4B). Rhizosphere microbiomes, even though the sampling time was at the inflorescence emergence stage, were mainly influenced by the soil (mean identity bacteria, 12.2%; mean identity fungi, 21.6%), while only minor fungal traits from the seed were found in the rhizosphere (mean bacteria, 1.2%; mean fungi, 11.5%). Interestingly, major differences of microbial community linkage between genotypes were observed. The main cultivar of organic Styrian oil pumpkin farming, GL Classic, showed the highest link of bacterial microbiome (60%) from sown seed to progeny seed, while almost no fungal traits were linked (0.7%). Especially the two cross-hybrids Gl. Diamant (A × B) and GL Rustikal [(A × B) × D] showed, in comparison to other genotypes, a higher linkage of fungal traits from sown seed to progeny seed [10.2% (Gl. Diamant) and 19.6% (GL Rustikal)]. Moreover, both showed, in addition to line B, a higher linkage of the fungal microbiome in sown seeds and the rhizosphere [16.6% (Gl. Diamant), 18.7% (GL Rustikal), and 16.3% (line B)]. Interestingly, the weakest link for bacterial and fungal strains from soil to rhizosphere (9.8 and 18.1%) was recorded for Gl. Diamant and the strongest link (15.1 and 24.4%) for GL Classic.

## Discussion

By performing an in-depth analysis of the bacterial and fungal communities associated with pumpkin plants throughout their life cycle and comparing multiple related genotypes, overall a strong genotype specificity of seed-associated microbial communities was observed. Besides habitat type (seed, rhizosphere, and soil), the genotype was an influential factor for differences between samples. Moreover, in accordance to previous studies, the seed microbiome, in comparison to the rhizosphere and soil microbiomes, has a lower diversity for the bacterial and fungal communities, with the highest diversity generally found in soil (Bulgarelli et al., 2012; Adam et al., 2018). In the rhizosphere, plants attract soil microbes with the release of plant root exudates, which often belong to plant-beneficial bacteria (Berendsen et al., 2012). In our study, we showed a higher abundance of *Burkholderiaceae*, *Rhizobiaceae*, and *Flavobacteriaceae* in the rhizosphere, which are bacterial families associated with plants and often harbor beneficial traits (Soltani et al., 2010; Carrión et al., 2018; Harman and Uphoff, 2019). Interestingly, in comparison to seeds, no genotype-specific differences in colonization were observed in the rhizosphere, even though the sampling time was at a stage where the plant was almost fully developed (BBCH:52). This could be due to plants being in the exact same soil or indicate that all pumpkin genotypes investigated in this study release similar root exudates; however, further investigations are needed to fully answer this question.

Genotype-specific seed microbial communities were discussed before and found for multiple plant species (Rybakova et al., 2017; Adam et al., 2018; Chen et al., 2020). Plant seeds were moreover discussed as carriers of plant-beneficial bacteria to the next generation (Bergna et al., 2018). In the course of breeding, the genetic traits of plants are selected, which possibly also change the microbiome, which is why a genotype-specific seed and rhizosphere microbiome can be observed (Adam et al., 2018; Wassermann et al., 2019). In addition, our results show that the soil in which the plants are cultivated has a severe effect on the microbiome of the next generation of seeds. This is also shown as the field origin and soil characteristics of sown seeds were shown to have a substantial influence on the observed beta diversity in our experiment. In our experiment, all seeds were planted in the same field to exclude this factor; however, additional experiments using other soil characteristics (e.g., different pH) could give a better indication of the severe soil effect. Interestingly, in accordance to Bergna et al. (2018), a high fraction of the bacterial microbiome of the sown seeds is linked to the progeny seeds. However, when looking at the fungal microbiome, a different picture was observed. Nevertheless, to fully disentangle the vertical transmission of seed-associated microbes, sequencing resolution on strain level is necessary. While only a low fraction of bacteria found in progeny seeds was linked to the soil and rhizosphere, for fungi this was the strongest linkage. This observation could be due to the fungicide treatment of seeds prior to sowing. Therefore, the assembly of fungal rhizosphere communities was mainly shaped by the soil and, to a lesser extent, by seed communities. The fact that the linkage of soil to rhizosphere was weak for Gl. Diamant and strong for GL Classic might be the first hint to a genotype-specific susceptibility to soil pathogens of GL Classic. Gl. Diamant expresses a very high tolerance to fruit rot, whereas GL Classic is the most susceptible genotype in the present study. Gl. Diamant is a genotype carrying resistance genes against ZYMV and is expressing a very high tolerance to that virus (AGES, 2020). A link between tolerance to ZYMV and some general pathogen recognition genes is discussed already (Capuozzo et al., 2017).

The generally high abundance of *Enterobacteriaceae* in seeds (sown seeds and progeny seeds) was remarkable. However, this was already reported in our previous study on pumpkin seeds (Adam et al., 2018) and found for other plants, e.g., tobacco, as well (Chen et al., 2020). Apart from *Enterobacteriaceae*, progeny seeds harbored higher abundances of *Bacillaceae*, *Pseudomonadaceae*, *Burkholderiaceae*, and *Sphingomonadaceae*. These groups, however only found in low abundances in sown seeds, are possibly enriched by the plant over the growing process due to their plant-beneficial traits (Mendes et al., 2013; Mandic-Mulec et al., 2016; Carrión et al., 2018). For instance, *Burkholderiaceae* and *Pseudomonadaceae* are known for their bioactive metabolite production, which has anti-fungal activity and influences plant growth (Kai et al., 2007; Thomashow et al., 2019). Furthermore, *Burkholderiaceae* and *Sphingomonadaceae* were found associated to plants in disease-suppressive soils (Chapelle et al., 2016). Our data indicate that plants could enrich specific microbes in their next-generation seeds that may play important roles in supporting the health, growth, and fitness of their hosts.

No differences regarding microbial diversity could be found between the group of homozygous inbred lines (lines A, B, and D), the single cross-hybrid (Gl. Diamant—heterozygous), the three-way cross-hybrid (GL Rustikal—heterozygous), and the population cultivar (GL Classic—heterozygote genome in certain loci), indicating that homozygote lines do not suffer from inbreeding depression regarding their microbial diversity. Moreover, high microbial linkages between breeding lines and hybrids were observed. The breeding lines and their hybrids shared an especially high fraction of their bacterial microbiome. The fungal microbiomes, however, were generally shared to a lesser extent. As noted previously, the fungal traits found in progeny seeds are mainly linked to the soil and rhizosphere. This could explain the weak linkage between fungal traits in sown seeds along the pedigree line since seeds originated from different fields. In contrast, the progeny seeds showed, similarly to the sown seeds, a generally lower linkage of fungal compared to bacterial traits. Since those seeds were grown in the same field, this could indicate that the transmission of fungal traits to the next generation is generally less directed, but more data are needed to fully explain this. Nevertheless, knowledge of the microbial composition of an inbred line or hybrid might be of high interest in the future, especially if more knowledge about which strains, transmitted from one generation to the next generation, in seed propagation are beneficial and which strains are potential pathogens is available. It could be worth knowing if a line tends to transmit more pathogens to the next generation or tends to attract more beneficial strains from soil than another line.

## Conclusion

The present study of pumpkin bacterial and fungal communities in seeds, rhizosphere, and progeny seeds showed a strong genotype specificity of bacteria in contrast to fungal communities in seeds. Bacterial traits are mainly linked from sown seeds to progeny seed, while fungal communities are mainly shaped by the soil and rhizosphere, which supports the understanding of seed microbiome assembly. Due to the selection of related pumpkin genotypes, our findings contribute to the new direction of microbiome-assisted plant breeding. With a targeted approach during plant breeding, not only desired plant traits but also healthy and plant-beneficial microbial communities on the seeds could be achieved.

## Data Availability Statement

All sequencing data supporting the findings of this study was submitted to the European Nucleotide Archive (ENA) and can be found under accession no. PRJEB41779 at www.ebi.ac.uk/ena.

## Author Contributions

EA, MB, HM, and GB designed the study. EA, MB, and HM performed the field experiments and prepared the samples for sequencing. PK, WW, and EO analyzed the data. PK, EA, WW, and GB wrote the manuscript. PK and EA contributed equally. All authors agreed on the final version of the manuscript.

## Conflict of Interest

EA and MB were employed by Saatzucht Gleisdorf GmbH. The remaining authors declare that the research was conducted in the absence of any commercial or financial relationships that could be construed as a potential conflict of interest.
