# Supplementary material for: Microbiome-Assisted Breeding to Understand Cultivar-Dependent Assembly in Cucurbita pepo
Source: Front Plant Sci. 2021 Apr 9;12:642027. doi: 10.3389/fpls.2021.642027 (PMC8063107; doi:10.3389/fpls.2021.642027)
Supplement: Supplementary file 1 [file Data_Sheet_1.docx]

# Supplementary material

Table S1: Sequencing results overview and observed alpha diversity in each sample.

| #SampleID | Sample type | Bacteria | | | Fungi | | |
| --- | --- | --- | --- | --- | --- | --- | --- |
|  |  | high quality read | target read | Shannon index | high quality read | target read | Shannon index |
| Pumpkin_KA003 | Progeny seed | 71295 | 3197 | 2.8 | 22578 | 293 | - |
| Pumpkin_KA015 | Progeny seed | 590375 | 240766 | 1.1 | 54331 | 358 | 2.2 |
| Pumpkin_KA023 | Progeny seed | 327372 | 68966 | 1.5 | 47955 | 836 | 2.5 |
| Pumpkin_KA033 | Progeny seed | 85240 | 4584 | 3.5 | 36902 | 452 | 2.5 |
| Pumpkin_KA002 | Progeny seed | 173263 | 4902 | 3.9 | 37169 | 466 | 2.2 |
| Pumpkin_KA014 | Progeny seed | 189420 | 14692 | 1.1 | 58061 | 368 | 1.6 |
| Pumpkin_KA022 | Progeny seed | 339442 | 40982 | 1.7 | 44606 | 352 | 1.8 |
| Pumpkin_KA032 | Progeny seed | 229376 | 7807 | 2.4 | 30463 | 375 | 2.6 |
| Pumpkin_KA008 | Progeny seed | 35888 | 235 | - | 60838 | 931 | 2.3 |
| Pumpkin_KA017 | Progeny seed | 448131 | 136 | - | 47258 | 2628 | 1.2 |
| Pumpkin_KA024 | Progeny seed | 145837 | 1146 | 1.6 | 17202 | 115 | - |
| Pumpkin_KA038 | Progeny seed | 368238 | 11626 | 1.4 | 63975 | 1852 | 0.9 |
| Pumpkin_KA009 | Progeny seed | 164642 | 98435 | 0.9 | 21243 | 1236 | 0.9 |
| Pumpkin_KA018 | Progeny seed | 150234 | 977 | 4.7 | 23866 | 4817 | 0.7 |
| Pumpkin_KA021 | Progeny seed | 191735 | 869 | 2.3 | 74190 | 1168 | 2.5 |
| Pumpkin_KA034 | Progeny seed | 341988 | 27979 | 1.2 | 15260 | 393 | 2.1 |
| Pumpkin_KA001 | Progeny seed | 606441 | 7752 | 2.2 | 64178 | 962 | 3.0 |
| Pumpkin_KA016 | Progeny seed | 354457 | 22707 | 0.8 | 86530 | 7469 | 2.4 |
| Pumpkin_KA031 | Progeny seed | 234104 | 2714 | 4.2 | 66087 | 1053 | 2.6 |
| Pumpkin_KA037 | Progeny seed | 550869 | 3429 | 4.2 | 64620 | 854 | 2.3 |
| Pumpkin_KA013 | Progeny seed | 420211 | 136944 | 0.9 | 32782 | 609 | 2.6 |
| Pumpkin_KA020 | Progeny seed | 214229 | 1218 | 3.4 | 52511 | 810 | 2.9 |
| Pumpkin_KA025 | Progeny seed | 98480 | 8184 | 3.6 | 35425 | 1840 | 2.6 |
| Pumpkin_KA035 | Progeny seed | 154050 | 2930 | 4.3 | 38646 | 367 | 2.2 |
| Pumpkin_KA065 | Rhizosphere | 458609 | 443668 | 5.0 | 91084 | 21148 | 2.7 |
| Pumpkin_KA066 | Rhizosphere | 920916 | 888094 | 5.7 | 85494 | 6528 | 3.3 |
| Pumpkin_KA067 | Rhizosphere | 129606 | 123742 | 5.6 | 95986 | 4789 | 3.4 |
| Pumpkin_KA068 | Rhizosphere | 469941 | 463092 | 5.3 | 84222 | 14480 | 3.1 |
| Pumpkin_KA069 | Rhizosphere | 561240 | 543716 | 5.4 | 91081 | 3588 | 3.2 |
| Pumpkin_KA070 | Rhizosphere | 765039 | 737976 | 5.5 | 62438 | 13826 | 2.9 |
| Pumpkin_KA071 | Rhizosphere | 408564 | 391016 | 5.4 | 39187 | 1803 | 2.9 |
| Pumpkin_KA072 | Rhizosphere | 651024 | 629000 | 5.4 | 66564 | 14867 | 3.0 |
| Pumpkin_KA073 | Rhizosphere | 1012994 | 958224 | 4.9 | 75452 | 5046 | 2.6 |
| Pumpkin_KA074 | Rhizosphere | 1001350 | 978640 | 5.3 | 67214 | 4579 | 2.9 |
| Pumpkin_KA075 | Rhizosphere | 893641 | 832250 | 5.6 | 48404 | 3025 | 3.3 |
| Pumpkin_KA076 | Rhizosphere | 1130433 | 1108928 | 5.0 | 63350 | 21173 | 2.9 |
| Pumpkin_KA077 | Rhizosphere | 406081 | 401878 | 5.2 | 88997 | 17510 | 2.8 |
| Pumpkin_KA078 | Rhizosphere | 143297 | 139298 | 5.3 | 74734 | 12568 | 2.5 |
| Pumpkin_KA079 | Rhizosphere | 139299 | 136122 | 5.2 | 77314 | 12062 | 2.9 |
| Pumpkin_KA080 | Rhizosphere | 457636 | 449347 | 5.5 | 45123 | 3357 | 3.1 |
| Pumpkin_KA081 | Rhizosphere | 183686 | 174595 | 5.1 | 91932 | 18343 | 2.8 |
| Pumpkin_KA082 | Rhizosphere | 315571 | 283213 | 5.5 | 38017 | 961 | 3.0 |
| Pumpkin_KA083 | Rhizosphere | 508436 | 492779 | 5.2 | 55909 | 4597 | 3.0 |
| Pumpkin_KA084 | Rhizosphere | 647659 | 640125 | 4.7 | 81715 | 33992 | 3.1 |
| Pumpkin_KA085 | Rhizosphere | 346859 | 333454 | 5.2 | 58521 | 8120 | 2.6 |
| Pumpkin_KA086 | Rhizosphere | 182315 | 175962 | 5.2 | 85679 | 10191 | 3.0 |
| Pumpkin_KA087 | Rhizosphere | 426075 | 413678 | 5.3 | 71463 | 7661 | 3.0 |
| Pumpkin_KA088 | Rhizosphere | 504765 | 486415 | 5.6 | 102647 | 17230 | 3.2 |
| Pumpkin_08sa | Sown seed | 52599 | 49712 | 2.3 | 9741 | 431 | 1.9 |
| Pumpkin_08sb | Sown seed | 12717 | 11454 | 2.5 | 30831 | 760 | 2.5 |
| Pumpkin_08sc | Sown seed | 16060 | 15467 | 1.7 | 51228 | 2236 | 2.5 |
| Pumpkin_08sd | Sown seed | 18482 | 17714 | 2.1 | 3839 | 338 | 1.9 |
| Pumpkin_09sa | Sown seed | 70014 | 67881 | 2.1 | 276047 | 52780 | 1.1 |
| Pumpkin_09sb | Sown seed | 23918 | 21346 | 1.6 | 36212 | 3016 | 1.8 |
| Pumpkin_09sc | Sown seed | 66050 | 62254 | 1.7 | 73933 | 10626 | 2.3 |
| Pumpkin_09sd | Sown seed | 67312 | 61435 | 1.7 | 99541 | 40114 | 0.7 |
| Pumpkin_01sa | Sown seed | 56864 | 55332 | 1.5 | 11080 | 3268 | 1.0 |
| Pumpkin_01sb | Sown seed | 48090 | 46380 | 1.5 | 3408 | 168 | - |
| Pumpkin_01sc | Sown seed | 41536 | 39057 | 1.5 | 53052 | 1367 | 2.3 |
| Pumpkin_01sd | Sown seed | 18169 | 17923 | 1.5 | 51276 | 1921 | 2.4 |
| Pumpkin_06sa | Sown seed | 71708 | 485 | 2.0 | 23342 | 1094 | 2.2 |
| Pumpkin_06sb | Sown seed | 50614 | 990 | 1.6 | 88127 | 3541 | 2.1 |
| Pumpkin_06sc | Sown seed | 27900 | 3560 | 0.9 | 32092 | 1346 | 2.3 |
| Pumpkin_06sd | Sown seed | 65056 | 2910 | 1.8 | 30184 | 1153 | 2.7 |
| Pumpkin_02sa | Sown seed | 48165 | 30163 | 2.1 | 93295 | 2428 | 2.4 |
| Pumpkin_02sb | Sown seed | 28494 | 22061 | 1.6 | 44699 | 734 | 2.4 |
| Pumpkin_02sc | Sown seed | 39902 | 34797 | 1.6 | 59168 | 154 | - |
| Pumpkin_02sd | Sown seed | 24706 | 18895 | 1.5 | 50822 | 1580 | 1.9 |
| Pumpkin_04sa | Sown seed | 19859 | 7792 | 1.8 | 32497 | 964 | 2.6 |
| Pumpkin_04sb | Sown seed | 11884 | 1789 | 1.6 | 14083 | 329 | 1.7 |
| Pumpkin_04sc | Sown seed | 96904 | 33800 | 1.8 | 9089 | 659 | 2.1 |
| Pumpkin_04sd | Sown seed | 115643 | 30912 | 1.7 | 112373 | 6083 | 2.1 |
| Pumpkin_KA117 | Soil | 167367 | 155248 | 6.0 | 143790 | 59433 | 3.7 |
| Pumpkin_KA118 | Soil | 319263 | 276652 | 5.9 | 124425 | - | - |
| Pumpkin_KA119 | Soil | 138248 | 128134 | 6.0 | 132549 | 63130 | 3.2 |
| Pumpkin_KA120 | Soil | 115342 | 106771 | 6.0 | 83263 | 40297 | 3.0 |

Table S2: Soil analysis of fields where the original seeds were produced.

| **Field** | **P_2_O_5_** | **K_2_O** | **Mg** | **B** | **Humus** | **K:Mg ratio** | **pH in CaCl_2_** |
| --- | --- | --- | --- | --- | --- | --- | --- |
| **Pfarrhoffeld** | *sufficient* | *sufficient* | *high* | *low* | *average* | *1.01* | *6.6* |
| **Teichacker** | *high* | *high* | *high* | *low* | *average* | *1.71* | *6.2* |


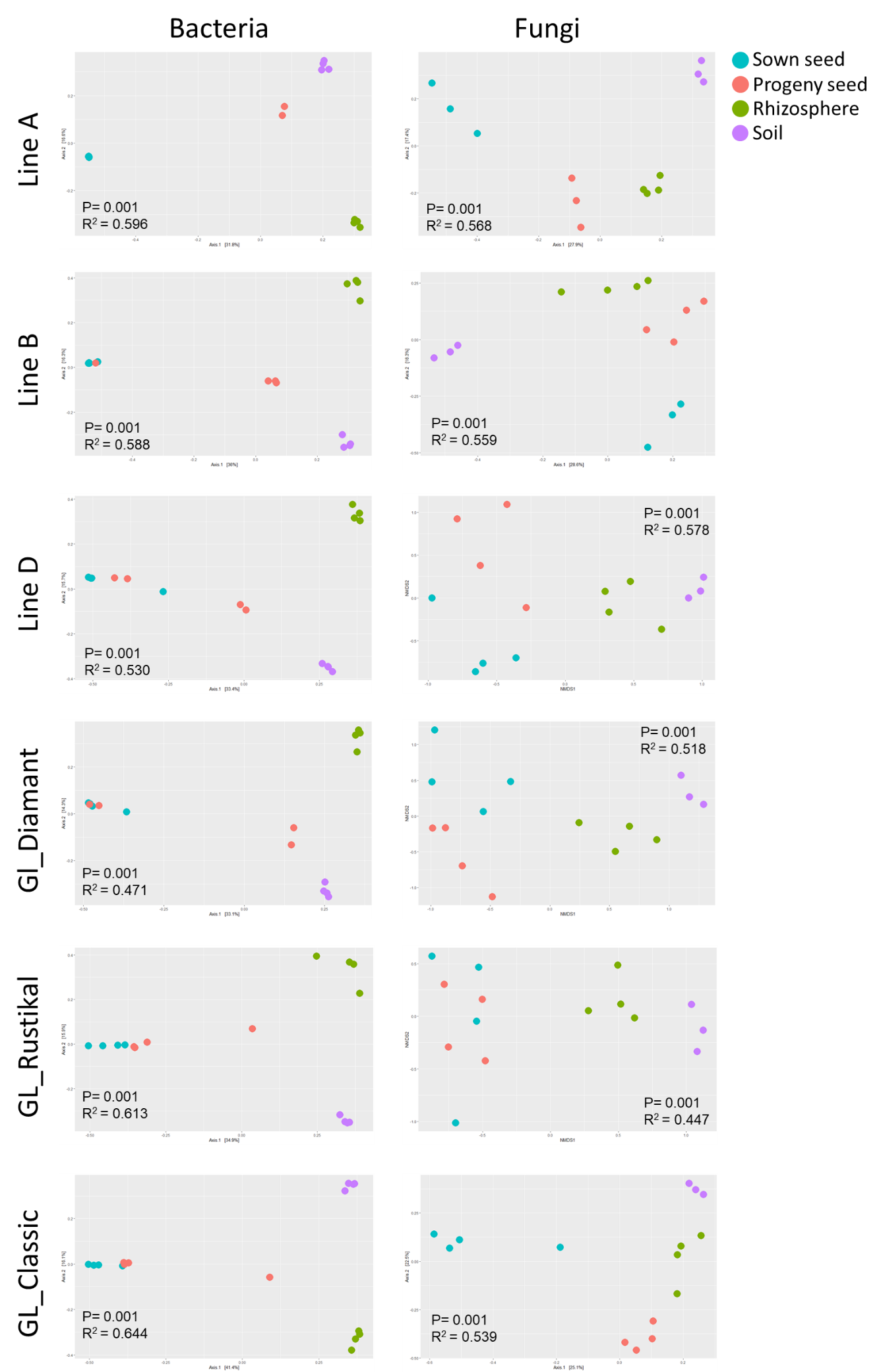


Figure S1: Observed beta diversity of each pumpkin genotype in seed, rhizosphere and soil.


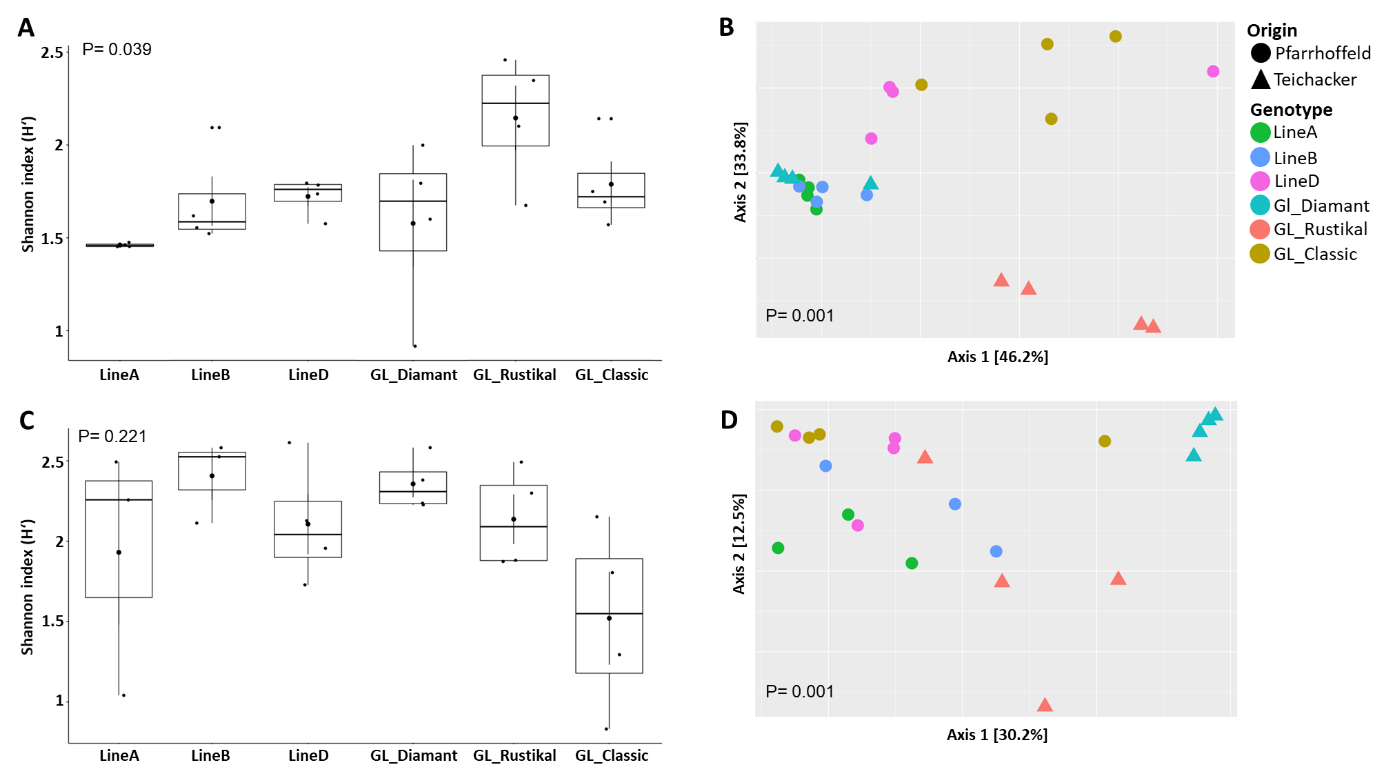


Figure S2: Observed alpha (A, C) and beta (B, D) diversity in the sown seed samples based on genotype (colors) and field origin (shapes). Bacterial (A, B) and fungal (C, D) diversities are shown.


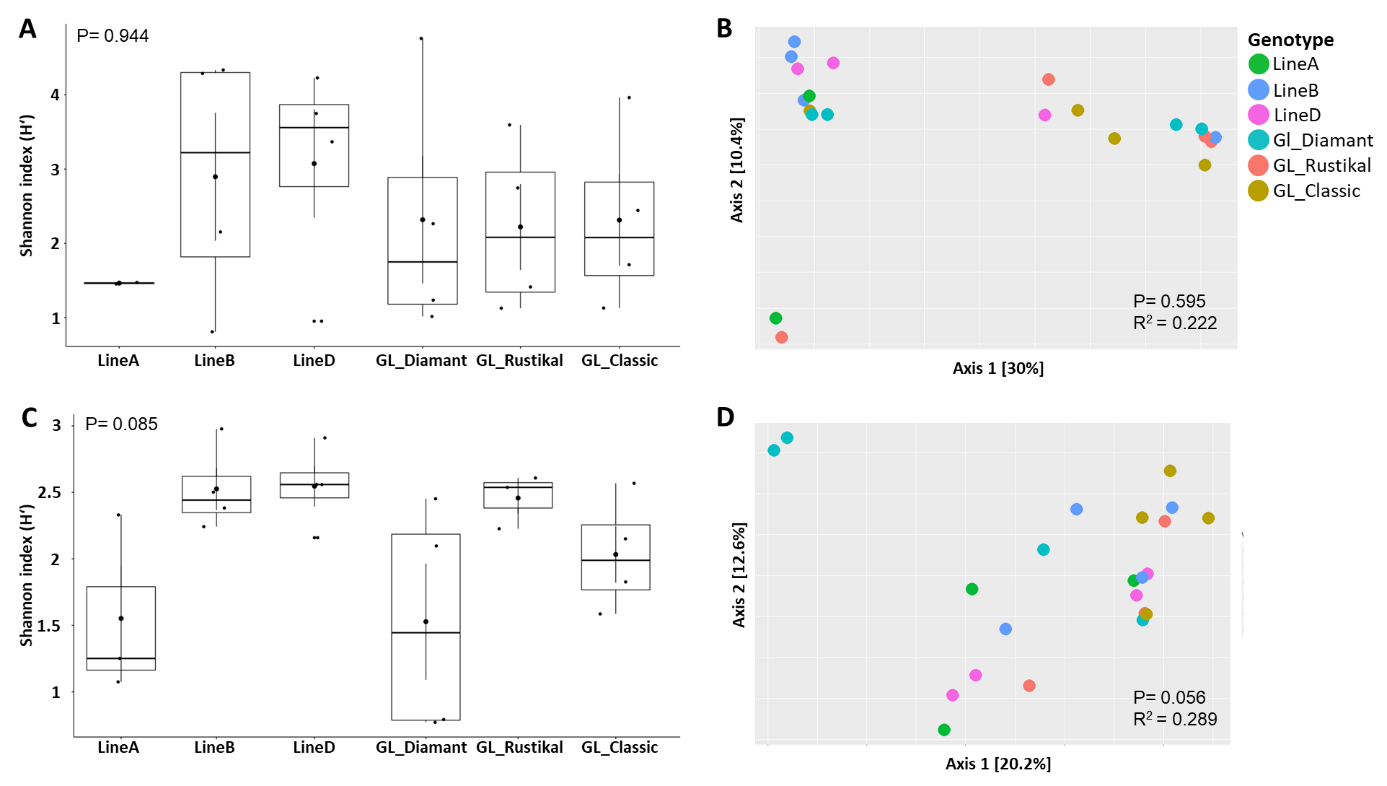


Figure S3: Observed alpha (A, C) and beta (B, D) diversity in the progeny seed samples based on genotype. Bacterial (A, B) and fungal (C, D) diversity is shown.


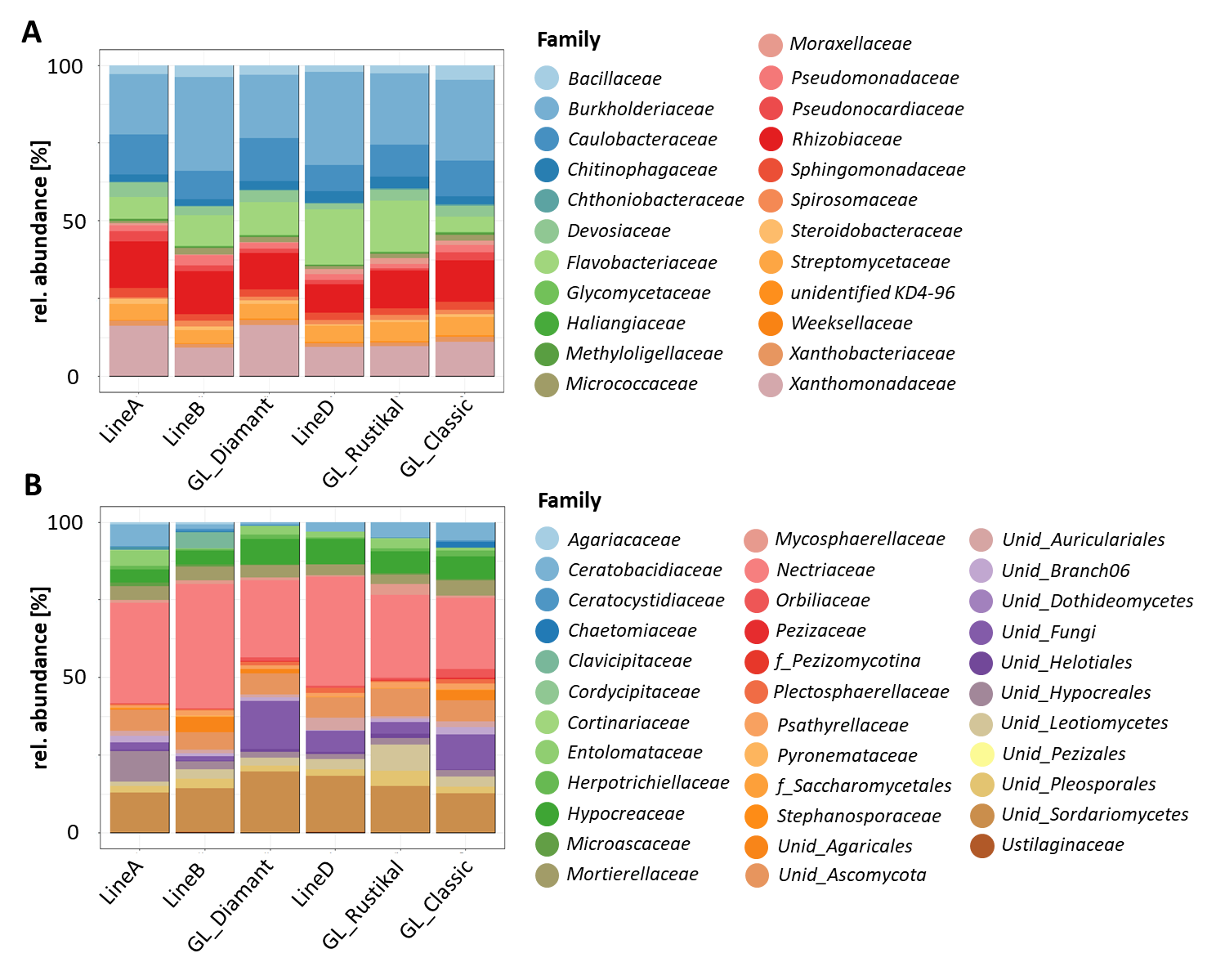


Figure S4: Relative taxonomic composition in the rhizosphere of different genotypes. Bacterial (A) and fungal taxonomic composition is shown on family level.
